# Expression of Cyclin D1 and Claudin-1 in Invasive Breast Carcinoma and Their Correlation with Clinicopathological Parameters

**DOI:** 10.30699/ijp.2024.2028643.3299

**Published:** 2024-10-02

**Authors:** Roopali Sehrawat, Vishesh Dhawan, Maitrayee Roy, Ayushi Kediya, Vijay Shrawan Nijhawan

**Affiliations:** 1 *Department of Pathology, SGT Medical College Hospital and Research Institute, SGT University, Gurugram, Haryana, India*; 2 *Department of Pathology, Maharishi Markandeshwar Institute of Medical Sciences and Research, Maharishi Markandeshwar Deemed to be University, Mullana-Ambala, Haryana, India*

**Keywords:** Breast cancer, Claudin-1, Cyclin D1, Immunohistochemistry

## Abstract

**Background & Objective:**

Evidence-based medicine has shown that patients with similar risk factors, stages, and therapy often have different clinical outcomes, highlighting the heterogeneity of breast cancer. In a quest for a better, cost-effective approach, researchers proposed the selection of surrogate IHC markers such as cyclin D1 and claudin-1 for the prognosis of breast cancer patients, supplementing the traditional ER, PR, and HER2/neu receptor.

**Methods:**

This retrospective study was conducted in a tertiary care hospital in northern India and included 50 cases of invasive breast carcinoma obtained from mastectomies, wide local excisions, and biopsies diagnosed over 4 years. In addition to ER, PR, and Her2/neu, claudin-1 and cyclin D1 IHC expression was assessed.

**Results:**

Cyclin D1 expression exhibited a statistically significant correlation with nodal status involvement (*P*=0.011) and with luminal-type breast carcinoma (*P*=0.023). Though there was no significant statistical correlation between claudin-1 and various clinic pathological features, it was seen to be positive in both luminal and HER2/neu-positive tumors.

**Conclusion:**

Our findings advocated the expression of IHC namely, cyclin D1 and claudin-1, in cases of breast cancer. Cyclin D1 positivity may show a significant association with better prognostic determinants while claudin-1 negative tumors may tend to be more often triple negative. Thus, IHC can be used in resource-constraint settings to substitute expensive molecular techniques.

## Introduction

With an increased incidence in younger age groups, breast carcinoma is rampant, both worldwide and in India, with an incidence rate of 23.8% and 19.5%, respectively, according to GLOBOCAN 2022 (1). Though having an almost 90% five-year survival rate in the Western world, it is quite dismal in India, where only three-quarters of women survive for 5 years (2). The majority of breast carcinomas are known to be sporadic with multifactorial etiology, including reproductive, lifestyle, and environmental factors. Among various genetic predisposing factors, BRCA1 and BRCA2 are known to have highest penetrance (3). Various markers have been developed to diagnose and prognosticate breast cancer patients including tumor size, histopathological grade, lymph nodal metastasis, and immunohistochemical (IHC) markers namely estrogen receptor (ER), progesterone receptor (PR) and HER2/neu. Evidence-based medicine has shown that patients with similar risk factors, stages, and therapy often have different clinical outcomes, highlighting the heterogeneity of breast cancer. Polyak suggested searching for more biomarkers that can help in better prognostication and faster prediction of therapy (4). Various molecular genomic tests on next-generation sequencing platforms, e.g., Oncotype DX, have been developed, although they remain a long shot for the masses in developing countries (5). Therefore, in a quest for a better cost-effective approach, researchers proposed the selection of various surrogate IHC markers namely cyclin D1, claudin-1, and epidermal growth factor receptor (EGFR) for prognosis of breast cancer patients, supplementing the traditional ER, PR, and HER2/neu receptor (6).

Cyclin D1 is known to control progression from G1 to S phase by regulating the action of cyclin-dependent kinases (CDKs) (6, 7). The binding of cyclin D1 to CDK4 and CDK6 helps in promoting cellular proliferation (8, 9). Thus, cyclin D1 overexpression is reported in around 50% of breast carcinoma. However, its effect on the behavior of breast cancer is still controversial (7, 8). Claudin, on other hand, maintains barrier functions between epithelial cells and cellular polarity. Several studies have indicated that reduced claudin expression, known as claudin low breast cancers, herald a poorer prognosis (10). We, hereby, assessed the IHC expression of cyclin D1 and claudin-1 in invasive breast carcinoma of women from a rural part of northern India and correlated with different clinicopathological variables.

## Materials and Methods

This retrospective study was conducted in a tertiary care hospital in northern India, catering to the rural population. We included 50 cases of invasive breast carcinoma obtained from mastectomies, wide local excisions, and biopsies diagnosed over 4 years from June 2015 to June 2019. Patients diagnosed with benign lesions and stromal tumors of the breast were excluded from the study. The histological diagnosis and the receptor (ER, PR, and HER2/neu) IHC slides were de-archived and re-evaluated by two pathologists. The pathological details included tumor size, histopathological tumor type, modified Bloom-Richardson grade, the status of nodal metastasis, and tumor stage wherever available. One formalin-fixed, paraffin-embedded tissue block processed from the tumor was selected in each case for further IHC staining with cyclin D1 (Clone RBT14, prediluted; Bio SB, Santa Barbara, CA, USA) and claudin-1 (polyclonal, prediluted; Bio SB, Santa Barbara, CA, USA) antibodies, along with appropriate positive controls, on Leica BOND-MAX IHC autostainer platform.

Cyclin D1 was interpreted as positive when ≥ 1% of invasive cancer cells exhibited nuclear staining of any intensity. The percentage of cyclin D1 positive tumor cells were noted and intensities were evaluated as weak, moderate, and strong. Claudin-1 was deemed positive when membrane staining of any intensity was observed in at least 1% of invasive tumor cells. Appropriate statistical analysis was performed using SPSS version V25. The clinicopathological characteristics were recorded in percentages and a chi-squared test for independence was used for correlations. A p-value of <0.05 was considered statistically significant. 

All procedures performed in the current study were approved by Institutional Ethical Committee Project No.: IEC-1052 dated 08/12/2017 in accordance with the 1964 Helsinki Declaration and its later amendments. Informed consent was obtained from all individual participants included in the study.

## Results

**Fig. 1:**
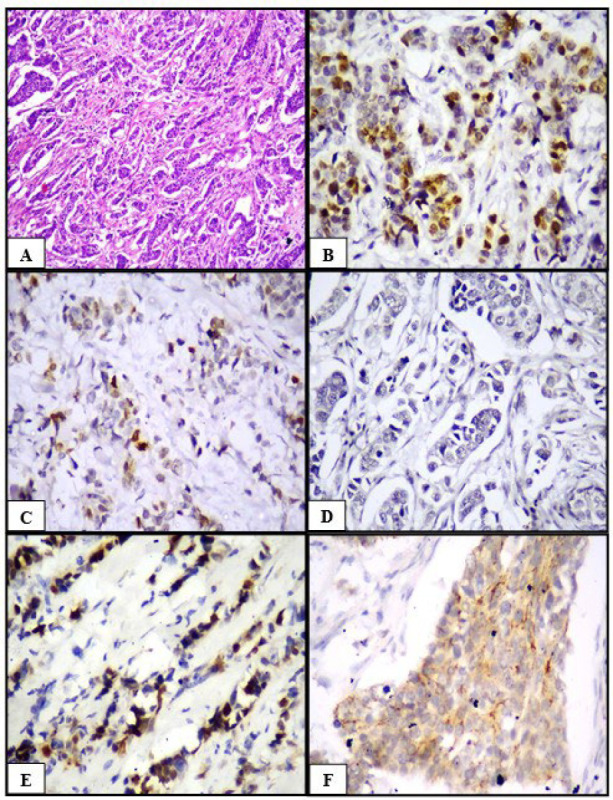
Invasive carcinoma NST (A) HPE (H&E, 100x), (B) ER positive (IHC, 400x), (C) PR positive (IHC, 400x), (D) HER2/neu negative (IHC, 400x), (E) Cyclin D1 positive (IHC, 400x), (F) Claudin 1 positive (IHC, 400x)

**Fig. 2:**
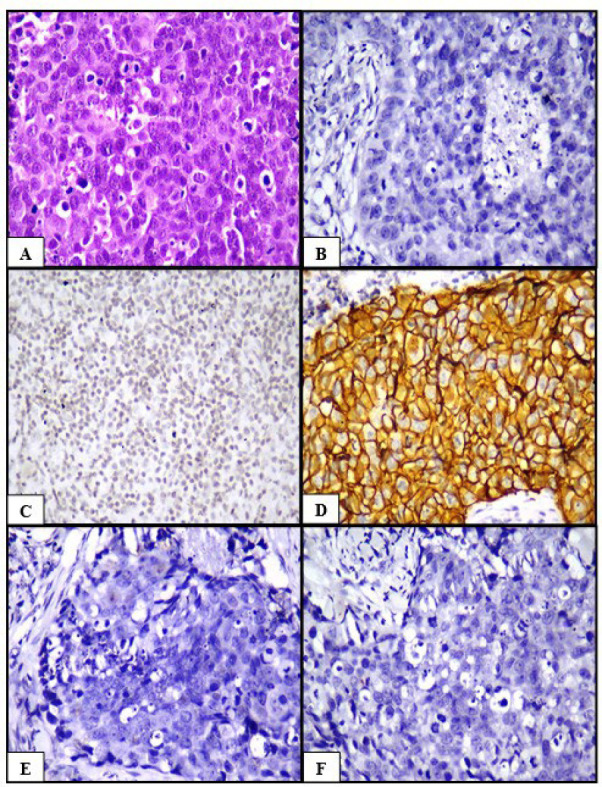
Invasive carcinoma, NST (A) HPE (H&E, 400x), (B) ER negative (IHC, 400x), (C) PR negative (IHC, 400x), (D) HER2/neu positive (IHC, 400x), (E) Cyclin D1 negative (IHC, 400x), (F) Claudin 1 negative (IHC, 400x)

**Fig. 3:**
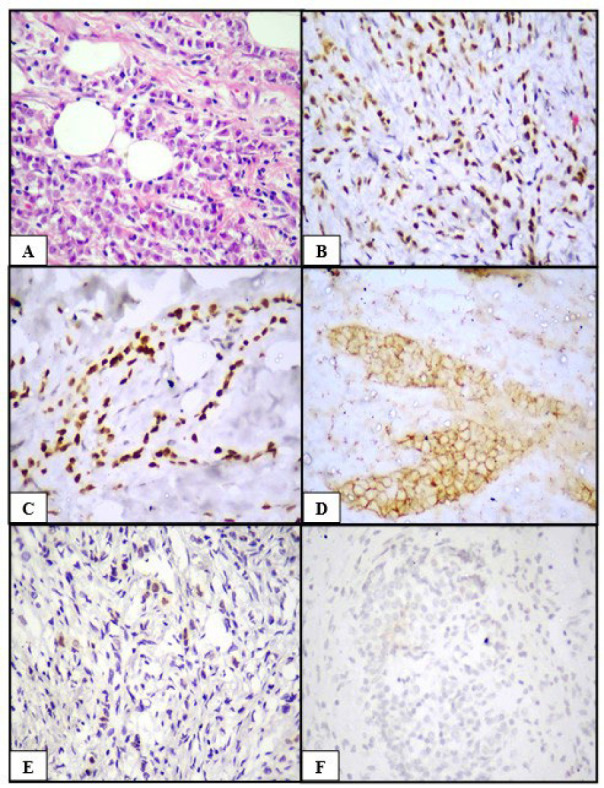
Invasive lobular carcinoma (A) HPE (H&E, 400x), (B) ER positive (IHC, 400x), (C) PR positive (IHC, 400x), (D) HER2/neu equivocal (IHC, 400x), (E) Cyclin D1 positive (IHC, 400x), (F) Claudin 1 negative (IHC, 400x)

**Fig. 4:**
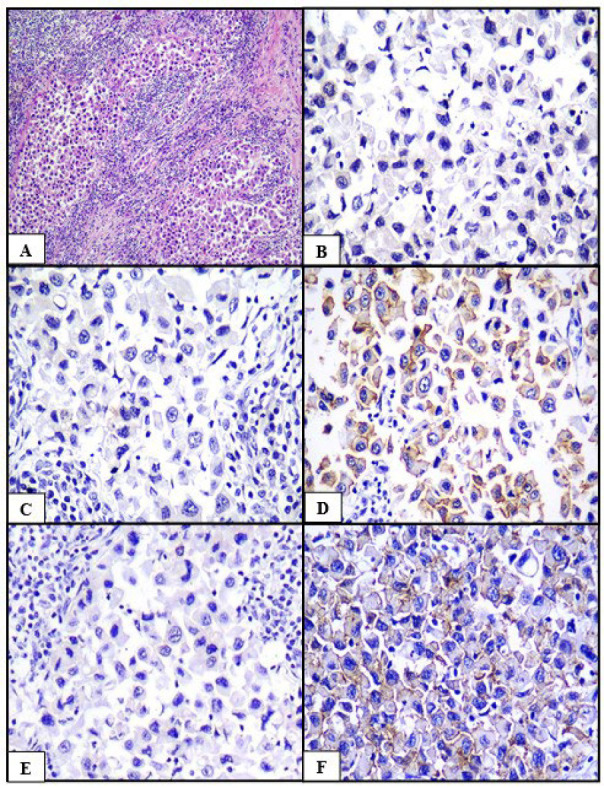
Carcinoma with medullary features (A) HPE (H&E, 100x), (B) ER negative (IHC, 400x), (C) PR negative (IHC, 400x), (D) HER2/neu positive (IHC, 400x), (E) Cyclin D1 negative (IHC, 400x), (F) Claudin 1 positive (IHC, 400x)

**Fig. 5:**
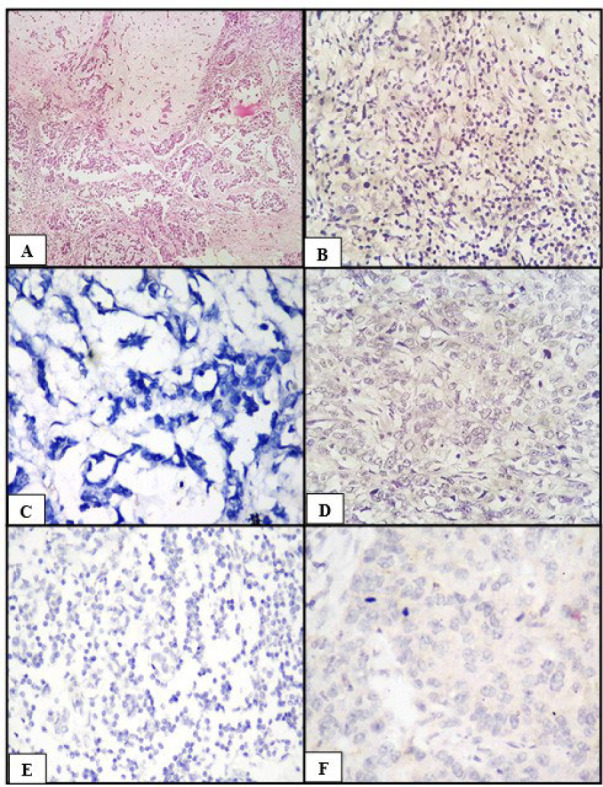
Metaplastic carcinoma (A) HPE (H&E, 100x), (B) ER negative (IHC, 400x), (C) PR negative (IHC, 400x), (D) HER2/neu negative (IHC, 400x), (E) Cyclin D1 negative (IHC, 400x), (F) Claudin 1 positive (IHC, 400x)

The study included 50 cases of breast carcinoma with a mean age of 51 years, age ranging from 35 to 85 years. Majority of the patients underwent mastectomy (80%), while 6 patients had widely metastatic disease at presentation and were deemed inoperable by the institute tumor board. Among the various clinicopathological variables, 78% of total patients aged >40 years with a mean tumor size of 4.8cm. The majority of the cases were conventional invasive carcinoma, NST constituting 84%, followed by invasive lobular carcinoma constituting 8%, metaplastic carcinoma, and carcinoma with medullary features (now known as tumor-infiltrating lymphocytes [TILs] rich invasive breast carcinoma as per 5^th^ edition WHO) constituting 4% of each of total cases (Figures 1, 2, 3, 4, and 5). Of the 37 patients who underwent mastectomies and lymph node dissection, 35.1% did not have any metastasis in axillary lymph nodes (pN0). Out of 24 patients with nodal metastasis, 32.4% of cases showed 1 to 3 positive nodes (pN1). 46% of patients belonged to triple-negative breast carcinoma strata. Regarding hormonal status, the majority were triple negative (46%) followed by luminal A (32%), HER2/neu overexpressing (16%), and luminal B (6%) tumors.

Cyclin D1 nuclear expression was observed in 38% of the cases, majorly belonging to > 40 years age group and having grade 2 invasive carcinoma (22%) ranging between 2 and 5 cm (pT2) in size (13 out of 44 patients). (Table 1) Cyclin D1 expression exhibited a statistically significant correlation with nodal status involvement (*P*=0.011) and with luminal-type breast carcinoma (*P*=0.023). Most cyclin D1-negative tumors did not express hormone receptors (ER and PR). They also tended to be more often negative for HER2/neu receptor. Table 2 depicts that 54.8% of cyclin D1 negative tumors were triple negative; however, this association was not statistically significant.

Claudin-1 expression was reduced in 74% of patients belonging to the>40 years age group (as seen in cyclin D1 expression) and those with grade 2 and grade 3 invasive carcinoma, measuring more than 2 cm in size. (Table 1) Both cases of carcinoma with medullary features had retained claudin-1 expression. Though there was no significant statistical correlation between claudin-1 and various clinic pathological features, claudin-1 was positive in both luminal and HER2/neu-positive tumors (Table 2).

**Table 1:** Correlation of Cyclin D1 and Claudin-1 tumors with the clinic-pathological features.

Parameters	Cyclin D1 Positive (n=19)	Cyclin D1 Negative (n=31)	P value	Claudin-1 Positive (n=13)	Claudin-1 Negative (n=37)	P-value
	(Allred score ≥2/8)	(Allred score <2/8)				
Age (n=50)						
≤40 years	4 (8%)	7 (14%)	0.899	3 (6%)	8 (16%)	0.913
>40 years	15 (30%)	24 (48%)		10 (20%)	29 (58%)	
Tumor size* (n= 44)						
≤ 2 cm	0 (0%)	3 (6.81%)	0.08	2 (4.54%)	2 (4.54%)	0.25
>2 and ≤ 5 cm	13 (29.54%)	12 (27.27%)		5 (11.36%)	19 (43.18%)	
>5 cm	4 (9.09%)	12 (27.27%)		6 (13.63%)	10 (22.72%)	
Histologic type (n=50)						
Invasive carcinoma, NST	15 (30%)	27 (54%)	0.287	11 (22%)	31 (62%)	0.05
Invasive lobular carcinoma	3 (6%)	1 (2%)		0 (0%)	4 (8%)	
Metaplastic carcinoma	1 (2%)	1 (2%)		0 (0%)	2 (4%)	
TILs-rich invasive breast carcinoma	0 (0%)	2 (4%)		2 (4%)	0 (0%)	
Histologic grade (n=50)						
Grade 1	1 (2%)	2 (4%)	0.681	0 (0%)	3 (6%)	0.473
Grade 2	11 (22%)	14 (28%)		6 (12%)	19 (38%)	
Grade 3	7 (14%)	15 (30%)		7 (14%)	15 (30%)	
Nodal status^#^ (n= 37)						
Nodal status not assessed (pNx)	6 (16.22%)	7 (18.92%)	0.011	2 (5.41%)	11 (29.73%)	0.23
Node – negative (pN0)	6 (16.22%)	7 (18.92%)		2 (5.41%)	11 (29.73%)	
1 to 3 nodes positive (pN1)	0 (0%)	12 (32.43%)		3 (8.10%)	9 (24.32%)	
4 to 9 nodes positive (pN2)	6 (16.22%)	3 (8.11%)		5 (13.51%)	4 (10.81%)	
≥10 nodes positive (pN3)	1 (2.70%)	2 (5.41%)		1 (2.70%)	2 (5.40%)	
ER status (n=50)						
Positive	11 (22%)	8 (16%)	0.023	4 (8%)	15 (30%)	0.532
Negative	8 (16%)	23 (46%)		9 (18%)	22 (44%)	
PR status (n=50)						
Positive	8 (16%)	4 (8%)	0.016	4 (8%)	8 (16%)	0.087
Negative	11 (22%)	27 (54%)		9 (18%)	29 (58%)	
HER2/neu status (n=50)						
Positive (score 3+)	4 (8%)	7 (14%)	0.299	4 (8%)	7 (14%)	0.625
Equivocal (score 2+)	4 (8%)	2 (4%)		1 (2%)	5 (10%)	
Negative (Score 0 to 1+)	11 (22%)	22 (44%)		8 (16%)	25 (50%)	

*Biopsies were excluded

^#^ Biopsies and simple mastectomies were excluded

**Table 2:** Association of Cyclin D1 and Claudin-1 tumors with the hormone receptor status

Hormone receptor status	Cyclin D1 Positive (n=19)	Cyclin D1 Negative (n=31)	p value	Claudin-1 Positive (n=13)	Claudin-1 Negative (n=37)	p value
Luminal A & B tumors	11 (57.8%)	8 (25.8%)	0.023	4 (30.8%)	15 (40.5%)	0.532
HER2/neu positive tumors	2 (10.6%)	6 (19.4%)	0.408	4 (30.8%)	5 (13.5%)	0.091
Triple negative tumors	6 (31.6%)	17(54.8%)	0.109	5(38.4%)	17 (45.9%)	0.526

## Discussion

Breast cancer is a malignancy in which individualized therapies have been successfully developed using molecular classification, resulting in marked improvements in disease-specific survival (DFS) (4). Using DNA microarrays and gene expression patterns, five different tumor subtypes have been described, namely luminal A, luminal B, HER2-enriched, basal-like, and claudin-low. A further ‘normal breast-like group’ has been identified, which is so named because this subtype expresses genes that are expressed by adipose tissue and other non-epithelial cell types, including basal epithelial genes (11). However, it is now considered to be an artificial category because of poorly sampled tumor tissue (12). Each subtype has distinct risk factors for incidence, response to therapy, risk of disease advancement, and different organs of metastasis (4).

Owing to demographic differences, the pathological characteristics of breast cancer occurring in Indian women are different from those observed in the Western world (13,14). The mean age of breast cancer reported in various parts of India ranges from 50 to 53 years, which was seen in concordance with our study (15-18). 

Testing for expression of ER, PR, and HER2/neu is now universally recommended in all invasive breast carcinoma patients that serve not only as prognostic biomarkers but also as predictive markers for determining adjuvant therapy (3, 19, 20). However, patients with the same risk factors and therapy have different outcomes, highlighting the heterogeneity of breast and necessitating the search for more biomarkers that can further personalize prognostication and prediction to therapy. (3) Molecularly, breast carcinoma is divided into luminal A, luminal B, HER2 enriched, basal-like, and claudin-low, based on DNA microarrays and gene expression patterns. (4) The luminal tumors are known to express ER and ER-related genes (SLC39A6, GATA3, FOXA1, and XBP1) along with GGH, LAPTM4B, CCNE1 seen additionally in luminal B tumors (21). HER2-enriched tumors are characterized by ERBB2 or HER 2 amplicon expression at chromosome 17q22.24, whereas basal-like tumors are p53 mutated and lack RB1 protein function. Claudin-low tumors lack cell-to-cell junction proteins, including E-cadherin (11). Triple-negative breast cancer (TNBC) is more prevalent in India (ranging from 27% to 35%) compared to Western countries (12% to 17%) (22). However, we found a higher prevalence of TNBC in 46% of our cases. 

Recent advances in genomics have led to the development of next-generation sequencing platforms, like Oncotype DX. This is a real-time polymerase chain reaction (PCR) based genomic test that helps quantify the risk of local recurrence and provides prognostic and predictive information in ER-positive lymph node-negative early-stage breast cancers. The test analyzes the expression of 21 genes, including 16 cancer-related and five control genes, to give a distant-disease recurrence score (RS) that ranges from 0 to 100 (12). These advances have assisted researchers in finding more biomarkers to personalize the prognosis and prediction to targeted therapy; these newer therapies still lie out of reach of most hospitals in India (5). IHC still remains the most widely available and cost-effective platform to test for newer molecular markers in developing countries. Cyclin D1 is known to promote cell proliferation by partnering with cyclin-dependent kinase 4 (CDK4) and/ or CDK6, which induces hyperphosphorylation of retinoblastoma (RB) protein and ultimately drives the G1 to S phase transition of the cell cycle. Claudin-1, on the other hand, is a tight junction transmembrane protein responsible for cell-to-cell adhesion and communication in epithelial and endothelial cells (23-25). It has been seen that cyclin D1 expression acts as a good prognostic and predictive determinant of breast carcinoma (26,27). Whereas claudin-1 expression is downregulated in many invasive breast carcinomas and tends to have an overall worse survival compared to luminal type (28).

Nottingham modification of the Scarff-Bloom-Richardson grading system was used to grade breast cancer cases. Half of the cases (50%) were grade 2, 44% were grade 3, and 6% were grade 1. We observed cyclin D1 overexpression in 38% of our patients with a statistically significant correlation between a lower incidence of lymph node metastasis and cyclin D1 expression (*P*=0.011), which was in concordance with studies by Guo *et al.* and Kucukzeybeket *et al.* (29,30). Most of the cyclin D1 positive cases had pT2 tumors, with the majority being grade 2 tumors and tumor size of >2 to </= 5 cm. Another statistically significant correlation was observed between the cyclin D1 expression and luminal-type breast carcinoma (*P*=0.023), which is in concordance with previous studies (29). Claudin-1, on the other hand, was reduced in 74% of patients. Claudin 1 positive case had a tumor size of >5 cm, belonged to invasive carcinoma NST and had 4 to 9 lymph nodes positive, although these findings were not statistically significant. 38.4% of claudin-1 positive tumors were seen in association with triple-negative breast cancers, being in discordance with Blanchard *et al.* (31). Hereby, our findings advocated the expression of IHC, namely, cyclin D1 and claudin-1, in cases of breast cancer. 

## Conclusion

Cyclin D1 positivity may show a significant association with better prognostic determinants, while claudin-1 negative tumors may tend to be more often triple negative. Thus, additional cyclin D1 and claudin-1 IHC can be used in resource-constraint settings to substitute expensive molecular techniques for better prognostication.

## References

[1] Sathishkumar K, Chaturvedi M, Das P, Stephen S, Mathur P (2022). Cancer incidence estimates for 2022 & projection for 2025: results from National Cancer Registry Programme, India. Indian J Med Res.

[2] Malvia S, Bagadi SA, Dubey US, Saxena S (2017). Epidemiology of breast cancer in Indian women. Asia Pac J Clin Oncol.

[3] Lakhani SR, Ellis IO, Schnitt SJ, Tan PJ, Van de Vijver MJ (2012). WHO Classification of Tumours of the Breast.

[4] Polyak K (2011). Heterogeneity in breast cancer. J Clin Invest.

[5] Wang M, Wu K, Zhang P, Zhang M, Ding A, Chen H (2019). The prognostic significance of the Oncotype DX Recurrence Score in T 1-2 N 1 M 0 estrogen receptor-positive HER2-negative breast cancer based on the prognostic stage in the updated AJCC 8th edition. Ann Surg Oncol.

[6] Gillett C, Fantl V, Smith R (1994). Amplification and overexpression of cyclin D1 in breast cancer detected by immunohistochemical staining. Cancer Res.

[7] Tobin NP, Sims AH, Lundgren KL, Lehn S, Landberg G (2011). Cyclin D1, Id1 and EMT in breast cancer. BMC Cancer.

[8] Ishii Y, Pirkmaier A, Alvarez JV (2006). Cyclin D1 overexpression and response to bortezomib treatment in a breast cancer model. J Natl Cancer Inst.

[9] Eeckhoute J, Carroll JS, Geistlinger TR, Torres-Arzayus MI, Brown M (2006). A cell-type-specific transcriptional network required for estrogen regulation of cyclin D1 and cell cycle progression in breast cancer. Genes Dev.

[10] Zhou B, Moodie A, Blanchard AA, Leygue E, Myal Y (2015). Claudin-1 in breast cancer: new insights. J Clin Med.

[11] Perou CM (2011). Molecular stratification of triple-negative breast cancers. Oncologist.

[12] Dabbs DJ (2014). Diagnostic Immunohistochemistry: Theranostic and Genomic Applications.

[13] Yang XR, Sherman ME, Rimm DL (2007). Differences in risk factors for breast cancer molecular subtypes in a population-based study. Cancer Epidemiol Biomarkers Prev.

[14] Bauer KR, Brown M, Cress RD, Parise CA, Caggiano V (2007). Descriptive analysis of estrogen receptor (ER) negative, progesterone receptor (PR) negative, and HER2 negative invasive breast cancer, the so-called triple negative phenotype. Cancer.

[15] Asthana S, Labani PS, Labani S (2015). A review on cancer incidence in India from 25 population-based cancer registries. J Dr NTR Univ Health Sci.

[16] Mohammadizadeh F, Hani M, Ranaee M, Bagheri M (2013). Role of cyclin D1 in breast carcinoma. J Res Med Sci.

[17] Peurala E, Koivunen P, Haapasaari KM, Bloigu R, Jukkola-Vuorinen A (2013). The prognostic significance and value of cyclin D1, CDK4 and p16 in human breast cancer. Breast Cancer Res.

[18] Ananthamurthy A, Ravikumar G (2014). Cyclin D1 expression in ductal carcinoma of the breast and its correlation with other prognostic parameters. J Cancer Res.

[19] Knight WA III, Livingston RB, Gregory EJ, McGuire WL (1977). Estrogen receptor as an independent prognostic factor for early recurrence in breast cancer. Cancer Res.

[20] Clark GM, Osborne CK, McGuire WL (1984). Correlations between estrogen receptor, progesterone receptor, and patient characteristics in human breast cancer. J Clin Oncol.

[21] Bhargava R, Dabbs DJ (2019). Diagnostic Immunohistochemistry: Theranostic and Genomic Applications.

[22] Sandhu GS, Erqou S, Patterson H, Mathew A (2016). Prevalence of triple-negative breast cancer in India: systematic review and meta-analysis. J Glob Oncol.

[23] Ko BS, Kim HJ, Yu JH (2013). Claudin-1, -3, -4, and -7 expression in triple-negative breast cancer. J Clin Oncol.

[24] Prat A, Parker JS, Karginova O (2010). Phenotypic and molecular characterization of the claudin-low intrinsic subtype of breast cancer. Breast Cancer Res.

[25] Xu J, Lan KT, Su TH (2017). Clinicopathologic and prognosis features of Claudin-low breast cancers. Zhonghua Bing Li Xue Za Zhi.

[26] Lundberg A, Lindström LS, Li J (2019). The long-term prognostic and predictive capacity of cyclin D1 gene amplification in 2305 breast tumours. Breast Cancer Res.

[27] Mohammadizadeh F, Hani M, Ranaee M, Bagheri M (2013). Role of cyclin D1 in breast carcinoma. J Res Med Sci.

[28] Tőkés AM, Kulka J, Paku S (2005). Claudin-1, -3, and -4 proteins and mRNA expression in benign and malignant breast lesions: a research study. Breast Cancer Res.

[29] Guo L, Liu S, Jakulin A, Yilamu D, Wang B, Yan J (2015). Positive expression of cyclin D1 is an indicator for the evaluation of the prognosis of breast cancer. Int J Clin Exp Med.

[30] Kucukzeybek BB, Bayoglu IV, Kucukzeybek Y (2017). The prognostic significance of cyclin D1 expression in patients with triple-negative breast cancer. J BUON.

[31] Blanchard AA, Ma X, Dueck KJ (2013). Claudin 1 expression in basal-like breast cancer is related to patient age. BMC Cancer.

